# Using human rights to advance global health justice in an age of inequality

**DOI:** 10.1371/journal.pgph.0003449

**Published:** 2024-07-09

**Authors:** Alicia Ely Yamin

**Affiliations:** Harvard Law School and Department of Health Policy and Management, Partners In Health, Harvard TH Chan School of Public Health, Boston, Massachusetts, United States of America; PLOS: Public Library of Science, UNITED STATES OF AMERICA

We are living in a world of vast inequality and an extreme concentration of wealth and power. By 2015, the richest 1% had as much wealth as the rest of the world’s population, and even as it wiped out livelihoods and pushed millions into poverty, the recent COVID-19 pandemic increased wealth at the top [1]. Gaping social inequality and the privatization of wealth and power undermine health equity and distort political agendas and multilateral governance around the world [2, 3]. The global economy more than doubled since 2000; however, neoliberal policies have led to the growth of private wealth, while public capital has declined in many countries [1, 3].

Such concentrated power in the hands of private actors allows them to control political agendas and shape laws [2–4]. Over the last few decades, investment, intellectual property, anti-trust, labor and tax laws, among others, have been mobilized in the service of unfettered economic growth for the few, while leaving extractivist pillaging of the environment and swathes of misery in their wake [3]. Globally, debt accumulation and unfair terms of exchange further deprive low- and middle-income countries of the resources they need to fund social endowments, including health [5]. It also translates into epistemic power to shape world-views and reify unfair knowledge practices in global health [6].

In this context, human rights offer a powerful counter-narrative that centers democratic pluralism and political equality. Human rights frameworks can undergird collective social mobilizations based not on the *sameness* that is rife in our increasingly tribalist world, but on shared commitments to equal dignity in all of our diversity.

In a recent book, I argue that to use human rights strategically moving forward, we first need to critically reflect on what we have achieved over the last thirty plus years to advance health rights, and where we have fallen short of our aspirations [3].

## Achievements and challenges in advancing health rights

Advancing health rights has called for understanding patterns of (ill-) health as the product of social, and power relations and the institutional arrangements, as much as biology or individual behavior [3]. When disparities in health are understood as misfortunes, they elicit lamentation, but when they are construed as rights violations and injustices, they call for redress [3].

Promoting health rights also calls for changes in international and national law. In different forms, affirmative entitlements to health care are now judicially enforceable across many countries, including very low-income countries, such as Uganda [7].

Likewise, tremendous progress has been made in advancing understanding of what applying human rights frameworks to public policy-making regarding health implies, such as multi-sectoral planning and budgeting, attention to disparities and marginalized groups from the outset and not as an afterthought, and a focus on social determinants of health [8].

These achievements have yielded far-reaching impacts not just in legislation, but in people’s well-being and lives in practice. Formularies have been changed, institutional practices altered, social discourses transformed, and budgets revised. Diverse populations have been protected by better regulations, received care they otherwise would not have, and appropriated an understanding of their own worth, dignity and full membership in their polities [3, 7].

At the same time, struggles to promote health rights have evolved in the context of transformations in global and national governance. The steady march of neoliberalism has changed the capacity of states to deliver on socio-economic rights, including health rights. For example, as of 2023, 85% of the world’s population is living under austerity, which has direct impacts on the capacity of states to guarantee health rights: 59 low- and middle-income countries are expected to spend less on health in 2024 than their average expenditure during the 2010s [9].

Debt-fueled austerity is especially bad for women’s health and rights, from access to reproductive health care to expanding care-giving burdens at home [10]. Further, failures of state institutions to deliver on promises of health and other socio-economic rights fuel populist waves, where women and LGBTQI+ populations bear a disproportionate brunt of the backlash [11].

Shrinking fiscal space, coupled all too often with the state abdicating to private interests within and beyond its borders, has fundamentally distorted the possibilities of using traditional human rights strategies focused on curbing abuses of power by the state to enhance health and social equality [3].

## Where do we go from here?

The right to health, as are all rights, is ineluctably embedded in broader legal and economic architectures, which positivistic approaches fail to challenge and may obscure. For example, advocating for the right to access to medicines without challenging intellectual property regimes is a windfall for pharmaceutical companies [4]. Likewise, “international assistance and cooperation” needs to be massively scaled up—and democratized to enable all states, together with civil society, to have a voice. But focusing on development assistance without addressing the structural rules of the global political economy entrenches more than challenges the inequity imbricated in the status quo [3].

Advancing health rights in our current context requires putting human rights in the service of building democratic political economies that strengthen transformative political possibilities. Doing so, in turn, calls for creative campaigns to hold transantional corporations, private financial institutions and other private actors accountable, curb their outsized power, and shift public attitudes from adulation of the mega wealthy to condemnation of the policy failures they represent [3, 4].

Efforts to outline “human rights-based economies” offer some key principles for restructuring our institutions, in health and beyond [12]. There is also increasing recognition that financing the right to health calls for an ‘economics of health for all,’ underpinned by human rights principles [13].

In turn, democratic governance at the national level is inseparable from the global financial architecture and governance arrangements. In addition to the dogged pursuit of commitments to fairer intellectual property rules and deconcentrating production during ongoing negotiations of a pandemic accord, notable efforts to curb the power of private actors include: the Africa-led initiative to create a UN Tax Convention that would set minimum tax levels regardless of where a transnational corporation operates [14]; and civil society coalitions’ campaigns to secure fairer processes for sovereigns to restructure their external debts while addressing domestic health and other needs [15].

## Conclusions

We have repeatedly witnessed groups using rights to collectively achieve progress that seemed impossible—until it happened—from HIV/AIDS activism to reproductive justice movements in Latin America. The struggle for health justice is more important than ever in this context of climate change, assaults on women’s health rights, the rise of populism, and savage inequities. Advancing health justice in today’s era of inequality calls for integrating human rights frameworks and principles into broader social and political movements, and for transformations in legal frameworks beyond human rights law that structure our political economies at national and global levels.
